# Dynamic Links Between Caregiving Trajectories and Subjective Life Expectancy in Chinese Older Care Recipients

**DOI:** 10.1093/geroni/igaf122.1608

**Published:** 2025-12-31

**Authors:** Xin Sun, Zi Yan

**Affiliations:** Fudan University, Shanghai, Shanghai, China (People’s Republic); Waseda University, Tokyo, Tokyo, Japan

## Abstract

Subjective life expectancy (SLE), an individual’s self-assessed lifespan prediction shaped by health, psychological, and sociodemographic factors, serves as a critical metric for evaluating caregiving efficacy and long-term health trajectories; however, how longitudinal caregiving trajectories dynamically shape SLE among care recipients remains underexplored. Based on data from 3,350 adults aged 60 and above across four waves (2011, 2015, 2018, and 2020) of the China Health and Retirement Longitudinal Study (CHARLS), group-based multi-trajectory modeling (GBTM)was employed to identify distinct caregiving trajectory groups. Latent growth curve modeling (LGCM) was then applied to examine how these trajectory types were associated with longitudinal changes in SLE. Four types of care trajectories were identified through GBTM: Sustained-Independence, Spouse-Anchored Gradual Transfer, Multi-to-child Shift, and Multi-Source Intensification. Latent growth curve modeling revealed that all care-receiving groups had lower baseline SLE than the ‘Sustained-Independence Group’. While the ‘Sustained-Independence Group’ maintained the highest baseline SLE, the ‘Multi-to-child Shift’ and ‘Spouse-Anchored Gradual Transfer Group’ exhibited converging trajectories over time. Notably, despite its initially lowest SLE levels, the ‘Multi-Source Intensification Group’ progressively narrowed the disparity with the ‘Sustained-Independence Group’ across waves. Our findings suggest that care dependency is not inherently linked to reduced perceived future time but is instead mediated by care provision dynamics. The study highlights the need for integrated, embedded, and modular support systems to alleviate mortality anxiety associated with perceived life constraints, offering actionable insights for developing interventions to enhance care recipients’ sense of agency and future orientation.

